# Serum Nrf2 Levels in Severe Traumatic Brain Injury Patients and Its Significance in Predicting 28‐Day Mortality

**DOI:** 10.1002/brb3.70786

**Published:** 2025-11-21

**Authors:** Gongjian Yin, Yaoxing Mu

**Affiliations:** ^1^ Department of Emergency Medicine Nanjing Tongren Hospital, School of Medicine, Southeast University Nanjing Jiangsu China

**Keywords:** 28‐day mortality, inflammatory response, Nrf2, STBI

## Abstract

**Objective:**

The objective of this study was to examine the levels of serum Nrf2 in patients with severe traumatic brain injury (STBI) and assess its predictive value for 28‐day mortality.

**Methods:**

This study prospectively observed 252 patients with STBI who were admitted to our ICU between January 2018 and April 2024. Serum samples were collected within 24 h of admission, and levels of nuclear factor erythroid 2‐related factor 2 (Nrf2) and inflammatory cytokines were measured using ELISA. Additionally, the mortality rate within 28 days after admission to the ICU was recorded.

**Results:**

In this prospective observational study, based on the 28‐day mortality after admission to the ICU, the patients were categorized into two groups based on their outcomes: the survival group (*n* = 184) and the deceased group (*n* = 68). Compared to the survival group, the deceased group exhibited lower serum Nrf2 levels and higher IL‐6 and IL‐17 levels. Spearman correlation analysis revealed a negative correlation between serum Nrf2 levels and serum IL‐6, IL‐1β, and IL‐17 levels, while a positive correlation was observed between serum Nrf2 levels and GCS scores. The ROC curve indicated that serum Nrf2 could be used to predict the 28‐day mortality in patients with STBI. Finally, multivariate logistic regression analysis showed that serum Nrf2, IL‐6, and IL‐17 levels were independently associated with 28‐day mortality in STBI patients.

**Conclusion:**

In conclusion, we observed significantly lower serum Nrf2 levels in deceased STBI patients compared to the survival group. Additionally, Nrf2 could be used as a potential marker to predict 28‐day mortality in STBI patients.

## Graphical abstract

This study analyzed 252 patients with severe traumatic brain injury and found that serum Nrf2 levels were significantly lower in those who died within 28 days. These findings suggest that serum Nrf2 may serve as a valuable prognostic biomarker for predicting short‐term mortality in patients with severe traumatic brain injury.

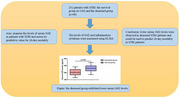

## 1 Introduction

Approximately 54.8 million people suffer from traumatic brain injury (TBI) each year, with a rate of 73 cases per 100,000 individuals. According to World Health Organization estimates, the majority of fatal injuries, about 90%, occur in low‐and middle‐income countries (Iaccarino et al. 2018). TBI is responsible for one‐third to one‐half of injury‐related deaths, making it a leading cause of disability in individuals under the age of forty (15–20 cases per 100,000 population annually). This has significant economic and societal implications, as patients require acute treatment, rehabilitation, and long‐term sequelae management, resulting in direct and indirect costs (Kamal et al. 2016; Capone‐Neto and Rizoli 2009; Dewan et al. 2018). Among TBI cases, severe traumatic brain injury (STBI) represents the most critical subset characterized by severe neurological impairment and high mortality rates (Robinson 2021). The pathophysiology of TBI involves both primary and secondary injury mechanisms. These include direct mechanical damage to edema, oxidative stress, and neuroinflammation (Kaur and Sharma 2018; Sulhan et al. 2020). Identifying potential markers that can predict the prognosis of TBI patients is urgently needed for effective management and therapeutic interventions.

Nrf2 is a critical transcription factor predominantly expressed in astrocytes, a major glial cell type involved in maintaining central nervous system (CNS) homeostasis (He et al. 2020; Iranshahy et al. 2018; Vargas and Johnson 2009). In the context of TBI, astrocytes play a dual role in both neuroprotection and the pathogenesis of secondary injury, particularly through their regulation of brain water balance and neuroinflammation. One of the key mechanisms is mediated by aquaporin‐4 (AQP4), a water channel protein primarily located on the perivascular endfeet of astrocytes. A study found that trifluoperazine reduced CNS edema and improved neurological recovery after spinal cord injury by inhibiting calmodulin‐dependent AQP4 membrane localization (Kitchen et al. 2020). Furthermore, trifluoperazine has also been shown to suppress AQP4 gene and protein expression in acute ischemic stroke, reducing cerebral edema and enhancing brain energy metabolism without adverse metabolic effects (Sylvain et al. 2021). These findings underscore the central role of astrocytes in TBI‐related edema and inflammation and suggest that astrocyte‐targeted pathways are promising therapeutic targets. Given that Nrf2 is highly expressed in astrocytes, its activation may reflect the astrocytic response to oxidative and inflammatory stress following TBI.

In addition to its well‐established role in cellular redox homeostasis, the activation of the Nrf2 signaling pathway has been shown to confer neuroprotection and mitigate neuronal damage in various neurological disorders (Kang 2020; T. Yang and Zhang 2021). In an animal study, promoting NRF2 expression could provide neuroprotection in a mouse model of TBI by inhibiting inflammatory responses (Wang et al. 2020). Although Nrf2 functions as a nuclear transcription factor, increasing evidence suggests that its presence in peripheral blood may result from injury‐induced glial activation, blood–brain barrier (BBB) disruption, and systemic inflammatory responses. During the acute phase of TBI, oxidative stress and cellular injury may trigger the release of intracellular proteins, including Nrf2, into the bloodstream. Therefore, serum Nrf2 levels may serve as an indirect indicator of CNS stress burden and glial response, rather than a direct marker of neuronal transcriptional activity. Compared to conventional prognostic markers such as the Glasgow Coma Scale (GCS), neuroimaging findings, and commonly studied serum biomarkers like S100B and NSE, Nrf2 may reflect a distinct pathophysiological axis—oxidative and inflammatory stress. Integrating Nrf2 into the current biomarker framework could enhance risk stratification and early mortality prediction in severe TBI. However, the role of Nrf2 in STBI and its potential as a short‐term prognostic biomarker for patients during their stay in the ICU remain largely unexplored.

Therefore, the purpose of this study is to investigate the serum levels of Nrf2 in STBI patients and assess its significance in predicting 28‐day mortality. We aim to contribute toward the development of novel therapeutic strategies and personalized interventions for improving the outcomes of STBI patients.

## 2 Methods

### 2.1 Subjects

This prospective observational study enrolled 252 STBI patients who were treated at our hospital from January 2018 to April 2024. The STBI patients were diagnosed with TBI based on computed tomography (CT) scans and were admitted to our hospital within 12 h of the trauma. These patients had a GCS score of ≤ 8. Exclusion criteria included (1) patients with severe infections in the past month, (2) patients with a history of severe head trauma, (3) patients with autoimmune diseases, (4) patients with neurological disorders, severe liver or kidney dysfunction, malignancies, or cardiovascular disorders, and (5) patients with severe traumatic injuries to other organs. None of the enrolled patients had a history of long‐term medication use prior to the injury. During ICU management, patients received standard supportive care including sedation, analgesia, and anticonvulsant therapy as needed. This study was approved by the Ethics Committee of Nanjing Tongren Hospital Affiliated to Medical College of Southeast University (approval number: 2018003), with approval granted in January 2018, covering the full study period from 2018 to 2024. All procedures involving human participants were conducted in accordance with the Declaration of Helsinki. Written informed consent was obtained from all participants or their legal guardians.

### 2.2 Treatment and Prognosis

We did not intervene in the treatment of patients in the research, and all patients admitted to our ICU received standard care based on their type and severity of injury. All patients underwent CT scans upon arrival at the emergency department and had intracranial pressure monitors placed. Patients with surgical lesions were urgently transferred to the operating room. Sedatives and analgesics were used for all STBI patients. Cerebrospinal fluid drainage, mannitol, and/or hypertonic saline were used as needed to reduce intracranial pressure. Apart from treatment methods implemented to control intracranial pressure (such as mannitol, craniotomy, etc.), there were no differences in the treatment approaches for all patients. Additionally, the survival status of all patients admitted to the ICU for 28 days was recorded.

### 2.3 Serum Biomarkers Assay

Peripheral venous blood samples (5 mL) were collected once from all patients within 24 h after injury. All samples were obtained during the early acute phase following trauma and prior to any major clinical interventions. Enzyme‐linked immunosorbent assay (ELISA) was subsequently performed to measure levels of Nrf2, interleukin (IL)‐6, IL‐1β, IL‐17, and C‐reactive protein (CRP) in the serum of all participants. Samples were centrifuged at 2000 *g* for 15 min, and commercially available assay kits were used for the measurements (CRP MBS8123937, MyBioSource, detection range: 54.69–3500 pg/mL, LoQ: 21.78 pg/mL, intra‐assay CV < 15%, inter‐assay CV < 15%; IL‐6 MBS2019894, detection range: 0.78–50 pg/mL, LoQ: 0.31 pg/mL, intra‐assay CV < 10%, inter‐assay CV < 12%; IL‐1β MBS3803011, detection range: 50–800 pg/mL, LoQ: 1 pg/mL, intra‐assay CV < 15%, inter‐assay CV < 15%; IL‐17 MBS2020546, detection range: 15.6–1000 pg/mL, LoQ: 5.6 pg/mL, intra‐assay CV < 10%, inter‐assay CV < 12%; Nrf2 MBS024775, MyBioSource, detection range: 62.5–2000 pg/mL, LoQ: 10 pg/mL, intra‐assay CV < 15%, inter‐assay CV < 15%; California, USA). According to the manufacturer's instructions, these kits have been validated for use in human serum.

### 2.4 Clinical Parameters Collection

Age, body mass index (BMI), gender, time from injury to hospital admission, and cause of injury were collected for all study participants. Additionally, the GCS was used for assessment upon hospital admission.

### 2.5 Statistical Analysis

Data analysis was performed using SPSS 26.0 (IBM, Armonk, NY, USA). Data were tested for normality using the Shapiro–Wilk test. Normally distributed variables are presented as mean ± standard deviation (SD) and compared using Student's *t*‐test, while non‐normally distributed variables are expressed as median (interquartile range, IQR) and compared using the Mann–Whitney *U* test. Proportions were analyzed using the chi‐square test. Spearman correlation analysis was performed to assess the associations between Nrf2 and other cytokines. Receiver operating characteristic (ROC) curve analysis was performed to evaluate the predictive value of serum Nrf2 levels for prognosis in STBI patients. Multivariate logistic regression analysis was performed to identify independent predictors of 28‐day mortality. Covariates included in the model were age, sex, BMI, time from injury to hospital admission, GCS score, CRP, IL‐6, IL‐1β, IL‐17, and Nrf2.

## 3 Results

### 3.1 Basic Characteristics

This prospective observational study included 252 patients with STBI. Based on the 28‐day mortality rate in the ICU, the participants were categorized into two groups based on their outcomes: the survival group (*n* = 184) and the deceased group (*n* = 68). In the survival group, the mean age was 38.33 ± 11.54, 47.8% were female, 55.4% had injuries due to motor vehicle accidents, and 25.0% had injuries due to falls. In the deceased group, the mean age was 37.62 ± 9.36, 52.9% were female, 52.9% had injuries due to motor vehicle accidents, and 20.6% had injuries due to falls. As shown in Table 1, there were no significant differences in age, sex, BMI, or time from injury to hospital between the deceased and survival groups. The GCS score was significantly lower in the deceased group, with a median of 4 (IQR 2), compared to 6 (IQR 3) in the survival group (*p *< 0.05).

**TABLE 1:** Basic characteristics of all study participants.

Variable	Deceased group, *n* = 68	Survival group, *n* = 184	*p*
Age, years	37.62 ± 9.36	38.33 ± 11.54	0.711
Sex, female (%)	36 (52.9)	88 (47.8)	0.471
BMI	22.56 (3.91)	22.49 (3.57)	0.759
Time from injury to hospital (h)	4.5 (6.25)	6 (5.75)	0.422
Causes of trauma			
Traffic accident, *n* (%)	36 (52.9)	102 (55.4)	0.781
Fall, *n* (%)	14 (20.6)	46 (25.0)	0.458
Other, *n* (%)	18 (26.5)	36 (19.6)	0.247
GCS	4 (2)	6 (3)	< 0.001

### 3.2 Expression of Nrf2 in Patients With STBI

**FIGURE 1:**
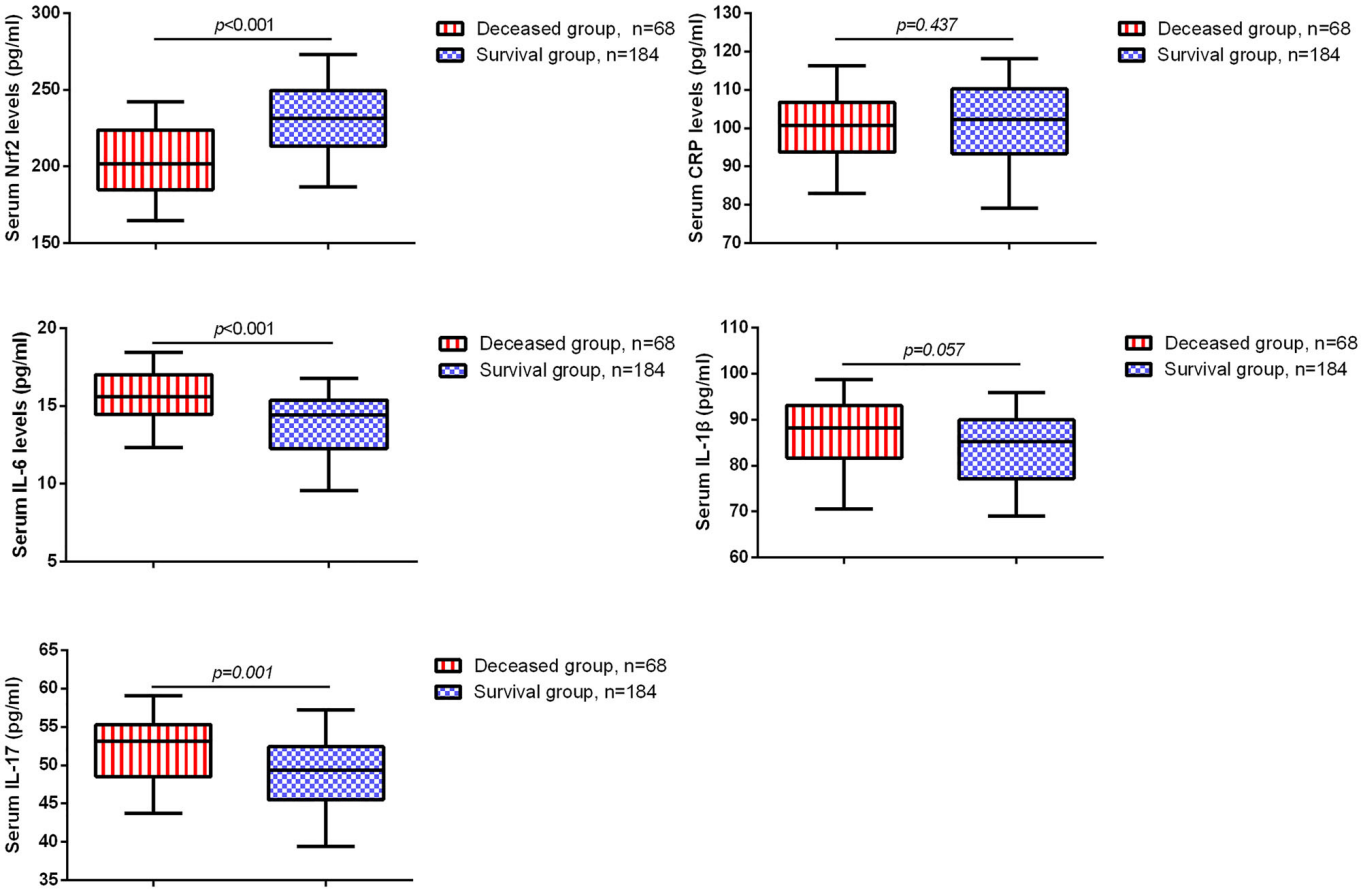
The expression of serum biomarkers in STBI patients.

Subsequently, we compared the levels of serum biomarkers in the two groups of STBI patients. As shown in Figure 1, the serum Nrf2 level was significantly lower in the deceased group (201.66 ± 22.09 pg/mL) than in the survival group (229.31 ±21.88 pg/mL). In contrast, IL‐6 and IL‐17 levels were significantly higher in the deceased group, with median values of 15.60 pg/mL (IQR 3.51) and 53.13 pg/mL (IQR 6.83), respectively, compared to 14.41 pg/mL (IQR 3.14) and 49.37 pg/mL (IQR 6.92) in the survival group. Spearman correlation analysis revealed a negative correlation between serum Nrf2 levels and serum IL‐6, IL‐1β, and IL‐17 levels, while a positive correlation was observed between serum Nrf2 levels and GCS scores (Table 2).

**TABLE 2:** Correlation analysis among Nrf2 and other inflammatory factors.

	Nrf2	CRP	IL‐6	IL‐1β	IL‐17	GCS
Nrf2						
Spearman's correlation	1	−0.043	−0.210	−0.176	−0.205	0.328
*p*		0.629	0.019	0.048	0.021	< 0.001
CRP						
Spearman's correlation	−0.043	1	−0.028	−0.001	0.081	0.033
*p*	0.629		0.754	0.990	0.365	0.717
IL‐6						
Spearman's correlation	−0.210	−0.028	1	−0.037	0.047	−0.037
*p*	0.019	0.754		0.682	0.601	0.682
IL‐1β						
Spearman's correlation	−0.176	−0.001	−0.037	1	0.035	−0.037
*p*	0.048	0.990	0.682		0.696	0.682
IL‐17						
Spearman's correlation	−0.205	0.081	0.047	0.035	1	−0.232
*p*	0.021	0.365	0.601	0.696		0.009
GCS						
Spearman's correlation	0.328	0.033	−0.037	−0.037	−0.232	1
*p*	< 0.001	0.717	0.682	0.682	0.009	

### 3.3 Predictive Value of Nrf2 for 28‐Day Mortality in Patients With STBI

**FIGURE 2:**
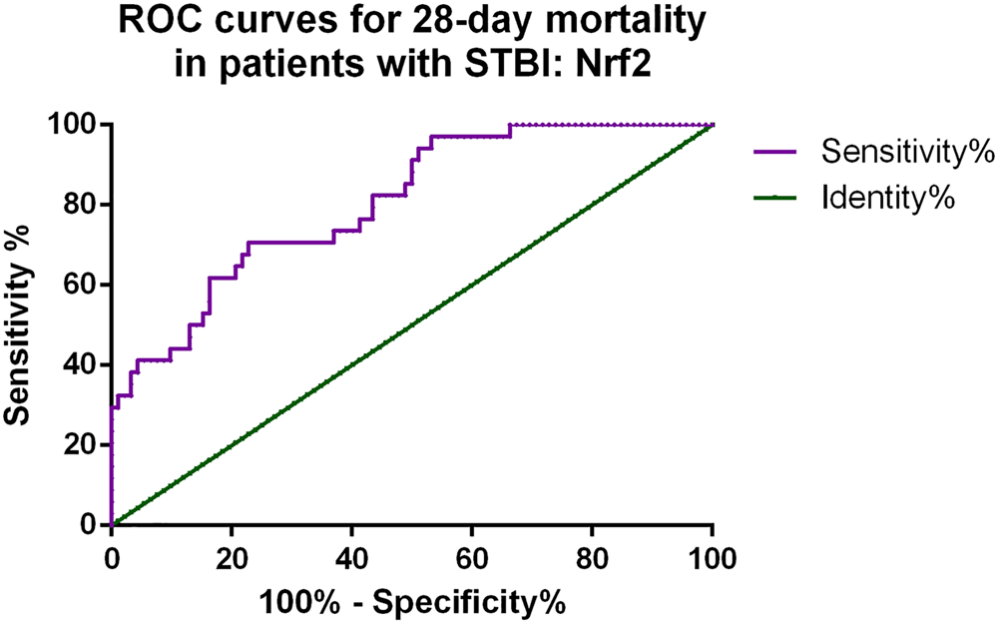
ROC curves of Nrf2 for 28‐day mortality in patients with STBI.

Furthermore, we conducted ROC curve analysis to assess the predictive value of serum Nrf2 levels for the 28‐day mortality rate in patients with STBI. As shown in Figure 2, the area under the ROC curve (AUC) for Nrf2 was 0.805 (95% CI: 0.723–0.887, *p* < 0.001), indicating good discriminative ability. The optimal cutoff value was 210.88 pg/mL, which yielded a sensitivity of 78.3% and a specificity of 67.6%.

### 3.4 Relationship Between Nrf2, Inflammatory Response, and 28‐Day Mortality in Patients With STBI

Subsequently, based on the ROC‐derived optimal cutoff value of 210.88 pg/mL, all STBI patients were stratified into a low Nrf2 group (*n* = 86) and a high Nrf2 group (*n* = 166). The results in Table 3 showed that patients in the low Nrf2 group had significantly lower GCS scores and remarkably elevated levels of serum IL‐6 compared to patients in the high Nrf2 group. Additionally, the 28‐day mortality rate was remarkably higher in the low Nrf2 group compared to the high Nrf2 group.

**TABLE 3:** Clinical characteristics of STBI patients between low/high Nrf2 levels.

Variable	Low Nrf2 group, *n* = 86	High Nrf2 group, *n* = 166	*p*
Age, years	35 (19)	38 (17)	0.845
Sex, female (%)	38 (44.2)	86 (51.8)	0.282
BMI	22.25 ± 2.36	22.60 ± 2.13	0.391
GCS	4 (2)	6 (3)	< 0.001
CRP (pg/mL)	101.43 ± 10.44	100.68 ± 10.13	0.697
IL‐6 (pg/mL)	15.07 ±2.32	14.10 ± 1.96	0.015
IL‐1β (pg/mL)	87.27 (12.96)	85.23 (13.02)	0.205
IL‐17 (pg/mL)	50.68 ± 4.22	49.61 ± 4.69	0.183
28‐Day mortality, *n* (%)	46 (53.5)	22 (13.3)	< 0.001

### 3.5 Binary Logistic Regression Analysis of Risk Factors for 28‐Day Mortality in Patients With STBI

Finally, multivariate logistic regression analysis was conducted to identify independent predictors of 28‐day mortality in patients with severe TBI. As shown in Table 4, higher IL‐6 levels were significantly associated with an increased risk of death (OR = 2.139, 95% CI: 1.384–3.306, *p* = 0.001). Elevated IL‐17 levels were also positively associated with mortality (OR = 1.181, 95% CI: 1.017–1.372, *p* = 0.029). In contrast, higher GCS scores (OR = 0.498, 95% CI: 0.309–0.801, *p* = 0.004) and higher serum Nrf2 levels (OR = 0.953, 95% CI: 0.924–0.983, *p* = 0.002) were independently associated with lower odds of mortality.

**TABLE 4:** Risk factors for 28‐day mortality in patients with STBI.

Variables	Wald	Odds ratio	95% CI	*p*
Age	0.213	1.014	0.955–1.078	0.644
Sex	0.117	0.803	0.229–2.218	0.732
BMI	0.318	1.083	0.822–1.426	0.573
Time from injury to hospital	2.438	0.848	0.691–1.041	0.115
GCS	8.260	0.498	0.309–0.801	0.004
CRP	0.935	0.969	0.910–1.033	0.334
IL‐6	11.709	2.139	1.384–3.306	0.001
IL‐1β	0.992	1.044	0.959–1.137	0.319
IL‐17	4.776	1.181	1.017–1.372	0.029
Nrf2	9.370	0.953	0.924–0.983	0.002

## 4 Discussion

TBI is the leading cause of trauma‐related deaths and disabilities worldwide, including STBI, which poses significant public health challenges due to its devastating consequences (Mostert et al. 2022; de Ramirez et al. 2012). Exploring risk factors for mortality in STBI patients is urgently needed to provide personalized care and reduce the mortality rate. In this study, we found that lower serum Nrf2 levels were independently associated with an increased risk of 28‐day mortality in patients with STBI.

Several factors have been identified as potential risk factors for adverse outcomes or death in patients with STBI. These factors include initial GCS score, age, presence of hypotension or hypoxia, and the presence of intracranial hemorrhage or diffuse axonal injury (Qu et al. 2011; Freeman et al. 2008; Kim et al. 2018). Our findings are consistent with previous studies showing that lower GCS scores are associated with increased mortality in STBI patients. This suggests that the initial neurological status of patients upon admission is a crucial factor in determining their prognosis. In addition to clinical factors, serum markers have emerged as valuable tools in predicting outcomes in STBI patients in recent years. For example, Feng et al. found that S100A12 may be correlated with brain inflammation, and evaluating the concentration of S100A12 in serum helps in early prediction of prognosis in STBI patients (Feng et al. 2018). Acute serum IL‐1β and IL‐6 levels contribute to assessing post‐concussive symptoms and predicting cognitive outcomes in patients with mild TBI (Sun et al. 2019). A recent study also indicated that dynamic monitoring of lactic acid and serum, and cerebrospinal fluid NSE after STBI can assess the condition and predict prognosis (Lu et al. 2024). Similarly, our study revealed significantly lower serum Nrf2 levels and significantly higher serum IL‐6 and IL‐17 levels in the deceased group compared to the survival group. Additionally, multivariate logistic regression analysis showed that serum IL‐6, IL‐17, and Nrf2 levels were independently associated with 28‐day mortality in STBI patients. However, as this was an observational study, further mechanistic investigations are required to determine whether Nrf2 exerts a causal protective effect. Astrocytes, the predominant glial cells in the CNS, play multifaceted roles in both acute injury response and long‐term functional recovery following TBI. Recent studies have revealed that AQP4, abundantly expressed in astrocytic membranes, undergoes dynamic translocation via endocytic pathways in response to osmotic and hypoxic stress. In particular, the involvement of early and recycling endosomes and cytoskeletal remodeling has been demonstrated to regulate AQP4 surface abundance, highlighting the potential of targeting AQP4 trafficking to modulate brain edema (Markou et al. 2024). In addition, emerging evidence indicates that astrogliosis—a hallmark response of astrocytes to CNS injuries—may exert dual effects on the injured brain. While excessive or prolonged astrogliosis contributes to glial scar formation and secondary injury, context‐dependent astrocyte activation may also promote neuroprotection, edema control, and tissue regeneration through mechanisms such as angiogenesis and synaptic remodeling (Alhadidi et al. 2024). These complex and often opposing roles suggest that astrocyte‐targeted therapies should be carefully timed and tailored to the injury stage. Given that Nrf2 is primarily expressed in astrocytes and modulates their antioxidant and anti‐inflammatory responses, it may play a key role in mediating these context‐dependent outcomes.

Nrf2 is a crucial transcription factor that plays a vital role in defending cells against oxidative stress and inflammation. It regulates the expression of numerous antioxidant and detoxification enzymes, as well as anti‐inflammatory molecules, through the activation of antioxidant response element (ARE)‐dependent genes (L. Yang et al. 2020). In clinical studies, Li et al. found that Nrf2 protein levels correlated not only with disease activity but also with the degree of renal injury in patients with lupus nephritis (LN) (Li et al. 2024). Gümüş et al. demonstrated that infection with COVID‐19 in pediatric patients resulted in decreased Nrf2 levels, decreased total antioxidant status (TAS) levels, and increased total oxidant status (TOS) and oxidative stress index (OSI) levels, thus explaining the tissue damage that may be caused by COVID‐19 (Gümüş et al. 2022). Yan et al. indicated that serum Nrf2 levels were correlated with the severity as well as the long‐term (180‐day) prognosis of patients with STBI (Yan et al. 2022). However, no studies have focused on whether there is an association between serum Nrf2 levels and ICU 28‐day mortality in STBI patients. In this study, we found that among STBI patients admitted to the ICU for 28 days, serum Nrf2 levels were significantly lower in patients in the deceased group. While the exact mechanisms underlying the association between Nrf2 and TBI outcomes are not fully understood, it is likely that Nrf2's role in regulating oxidative stress and inflammation is involved. Oxidative stress and neuroinflammation have been recognized as key contributors to TBI pathology, and Nrf2 activation has been shown to mitigate these processes in various neurological disorders. Thus, lower serum Nrf2 levels were associated with poorer prognosis in patients with severe TBI (Jayaram and Krishnamurthy 2021; Zhang et al. 2021; Khan et al. 2019), although whether this reflects a causal relationship requires further investigation. Our study highlights the importance of considering both clinical factors and serum markers in predicting outcomes in severe TBI patients. The association between lower serum Nrf2 levels and increased mortality underscores the potential role of Nrf2 as a prognostic marker and therapeutic target in TBI.

It is important to acknowledge the limitations of our research. First, this was a single‐center study, which may limit the generalizability of the findings to broader populations. Multicenter validation is warranted. Second, our study focused on the 28‐day mortality rate as the primary outcome measure. While this is a commonly used endpoint in TBI research, it may not fully capture the long‐term functional and cognitive outcomes in these patients. Third, our study focused on the association between serum Nrf2 levels and inflammatory markers, but we did not investigate the underlying mechanisms driving these associations. Further mechanistic studies are warranted to elucidate the pathways through which Nrf2 and inflammatory factors interact in the context of severe TBI.

## 5 Conclusion

In summary, our study found that lower serum levels of Nrf2, IL‐6, and IL‐17 were independently associated with increased 28‐day mortality in patients with STBI. These findings suggest the potential importance of Nrf2 as a protective regulatory pathway in the acute phase of TBI. However, given the observational nature of the study and the limited sample size, further multicenter studies and mechanistic investigations are needed to validate these associations and explore their clinical relevance.

## Author Contributions

**Gongjian Yin**: writing–original draft, data curation, software, investigation, validation, formal analysis, resources, visualization, methodology. **Yaoxing Mu**: conceptualization, methodology, writing–review and editing, supervision, project administration, resources.

## Ethics Statement

The present study was approved by the ethics committee of Nanjing Tongren Hospital Affiliated to Medical College of Southeast University of Science and Technology.

## Conflicts of Interest

The authors declare no conflicts of interest.

## Data Availability

All data in this study can be obtained by a proper request from the authors.
